# Keap1-Nrf2 Pathway Regulates ALDH and Contributes to Radioresistance in Breast Cancer Stem Cells

**DOI:** 10.3390/cells10010083

**Published:** 2021-01-06

**Authors:** Dinisha Kamble, Megharani Mahajan, Rohini Dhat, Sandhya Sitasawad

**Affiliations:** Redox Biology Lab, National Centre for Cell Science (NCCS), Pune 411007, India; dinisha.kamble19@gmail.com (D.K.); meghanandgaon@gmail.com (M.M.); rohini.dhat@nccs.res.in (R.D.)

**Keywords:** BCSC, ALDH activity, fractionated dose of γ radiation, radioresistance, ROS, Nrf2, Keap1, miR200a, epithelial–mesenchymal transition (EMT)

## Abstract

Tumor recurrence after radiotherapy due to the presence of breast cancer stem cells (BCSCs) is a clinical challenge, and the mechanism remains unclear. Low levels of ROS and enhanced antioxidant defenses are shown to contribute to increasing radioresistance. However, the role of Nrf2-Keap1-Bach1 signaling in the radioresistance of BCSCs remains elusive. Fractionated radiation increased the percentage of the ALDH-expressing subpopulation and their sphere formation ability, promoted mesenchymal-to-epithelial transition and enhanced radioresistance in BCSCs. Radiation activated Nrf2 via Keap1 silencing and enhanced the tumor-initiating capability of BCSCs. Furthermore, knockdown of Nrf2 suppressed ALDH^+^ population and stem cell markers, reduced radioresistance by decreasing clonogenicity and blocked the tumorigenic ability in immunocompromised mice. An underlying mechanism of Keap1 silencing could be via miR200a, as we observed a significant increase in its expression, and the promoter methylation of Keap1 or GSK-3β did not change. Our data demonstrate that ALDH^+^ BCSC population contributes to breast tumor radioresistance via the Nrf2-Keap1 pathway, and targeting this cell population with miR200a could be beneficial but warrants detailed studies. Our results support the notion that Nrf2-Keap1 signaling controls mesenchymal–epithelial plasticity, regulates tumor-initiating ability and promotes the radioresistance of BCSCs.

## 1. Introduction

Radiotherapy (RT) is a critical factor of primary, adjuvant and palliative treatment for almost all kinds of cancers, including breast cancer. It alone is capable of lowering the 10-year risk of relapse by one half and reducing the 15-year risk of breast-cancer-related death [1]. Although profound benefits are achieved with RT due to its localized treatment, especially for ductal carcinoma and early invasive cancer, local control of the disease fails by 8–15% in radiotherapy-treated patients with advanced invasive tumors due to resistance and relapse of the tumor [2]. The reason for RT failure and the locoregional recurrence of breast cancer is the presence of a subset of radioresistant tumor cells, termed breast cancer stem cells (BCSCs), which show a difference in sensitivities to radiation [3,4,5]. Standard fractionated doses of radiation are sublethal for BCSCs as they typically evade radiation to develop innate or acquired resistance and establish tumor recurrence and metastasis, leading to the majority of cancer-related deaths. The molecular mechanisms that govern the emergence of aggressive radioresistance in BCSCs are yet unknown.

Low levels of reactive oxygen species (ROS) and enhanced ROS defenses appear to partially contribute to the adaptive tumor radioresistance in BCSCs [5,6,7]. Thus, the identification of underlying mechanisms and overcoming low ROS levels within BCSCs may be a useful method for improving radiation therapy. The transcription factor nuclear factor (erythroid-derived 2)-like 2 (Nrf2), the master regulator of antioxidant defense mechanisms, is a critical regulator of the redox balance. In the cytosol, Nrf2 activity is tightly regulated by two main inhibitors, Keap1 and GSK-3. The Neh2 and Neh6 domains of Nrf2 are the degron. While the Neh2 domain binds the E3 ligase adapter Keap1 that presents Nrf2 for ubiquitination to a CUL3/RBX1 complex, the Neh6 domain requires previous phosphorylation by GSK-3 to bind the E3 ligase adapter b-TrCP and subsequent ubiquitination by a CUL1/RBX1 complex [8]. Inactivation of either of these regulators due to oxidative or electrophilic stress stabilizes Nrf2, which then translocates to the nucleus and binds to antioxidant response elements (ARE) in the promoter region of target genes by the formation of a heterodimer with small Maf proteins. In the nucleus, Bach1 negatively regulates nuclear Nrf2 activity by competitive-binding with small Maf proteins [9] and thereby inactivates HO1 [10,11]. Previous studies have shown elevated levels of Nrf2 as a critical regulator of chemoresistance in CSC-enriched breast tumors [12,13] and the activation of Nrf2-associated antioxidant genes, such as HO1, NQO1, Prx1, etc., that contribute to radioresistance in other cancer cells [14]. Since BCSCs contain low levels of ROS and enhanced antioxidant defense [5], the role of the Nrf2 pathway in the radioresistance of BCSCs deserves further investigation. 

In this study, we observed an increase in ALDH activity, indicative of BCSCs with increased radioresistance, tumorigenesis, reduced apoptosis and the activation of signaling pathways, which promote mesenchymal–epithelial transition (MET) and migration. Additionally, enhanced tumorigenicity was observed after fractionated irradiation. Further investigation of the role of Nrf2 in radioresistance showed that Nrf2 and its associated genes HO1 and NQO1 were significantly increased after irradiation. The shRNA-mediated knockdown of Nrf2 expression led to a decrease in all of the above processes of radioresistance in BCSCs. The mechanism of Nrf2 activation was found to be regulated via Keap1 silencing, as we did not see any change in GSK-3β, as well as in Bach1, the negative regulator of Nrf2. We also did not find any change in the methylation status of the Keap1 promoter; however, a significant increase in the expression of miR200a was observed. This indicates that miR200a could be a possible mechanism of Keap1 silencing. This study provides evidence for the role of Nrf2 and its downstream genes and suggests mechanisms by which the Nrf2/Keap1 pathway induces radioresistance in BCSCs. Overall, the data indicate the contribution of ALDH^+^ cell population to radioresistance via the Nrf2-Keap1 axis, suggesting that targeting ALDH^+^ BCSC cell population with miR200a could be beneficial but warrants detailed studies.

## 2. Materials and Methods

### 2.1. Reagents 

Antibodies recognizing NANOG (D73G4), SOX2 (D6D9), KLF4 (D1F2), HO1 (D60G11), Vimentin (D21H3) and Keap1 (D6B12) were obtained from Cell Signaling Technology (Danvers, MA, USA). E-cadherin (610404) was obtained from BD Biosciences (San Jose, CA, USA). Nrf2 (ab-89443), SLUG (ab-27568), SNAIL (ab-53519) and Bach1 (ab-115210) were purchased from Abcam, (Burlingame, CA, USA) and NQO1 (sc-32793), BAX (sc-7480), BCL2 (sc-7382) were obtained from Santa Cruz Biotechnology (Dallas, TX, USA). Nrf2-targeting shRNA lentiviral particles (sc-37030-v) were obtained from Santa Cruz Biotechnology (CA, USA). Bovine serum albumin (BSA), fibroblast growth factor (FGF) and epidermal growth factor (EGF) were purchased from Sigma-Aldrich (St. Louis, MO, USA). ALDEFLUOR Kit (01700) was obtained from STEM CELL technologies (Vancouver, Canada). Nrf2 Transcription Factor Assay Kit (Colorimetric) (ab207223) was obtained from Abcam. Annexin-V-FITC (556419) was obtained from BD Biosciences (San Jose, CA, USA). 

### 2.2. Cell Culture and Irradiation

MCF-7 and MDA-MB-231 cells were obtained from the American Type Culture Collection (ATCC) and maintained in DMEM supplemented with 10% FBS (Gibco, MD, USA). Cells were irradiated at room temperature with a ^60^Co-γ rays laboratory irradiator (Gamma Chamber-5000, BRIT, Mumbai) at a dose rate of 2.163 Gy/min for the time required to obtain the prescribed dose. For fractionated doses of radiation, cells were irradiated with 2Gy for three consecutive days, and for an acute dose of 6Gy, cells were irradiated once on the last day of fractionated irradiation. Corresponding controls were mock irradiated [13]. Control and irradiated cells were further incubated for 24 h postirradiation.

### 2.3. Mammosphere Formation

After irradiation, mammospheres were formed using single-cell suspensions in an ultralow attachment 6-well plate at a density of 2 × 10^4^ cells/well in specific media for mammosphere culture containing DMEM and Nutrient Mixture F-12 medium supplemented with 20 ng/mL EGF, B27 (1:50, Life Technologies, MA, USA), 20 ng/mL FGF (Sigma-Aldrich, St. Louis, MO, USA) and penicillin/streptomycin (Invitrogen) for 4–5 days. The floating aggregates with a >50 μm diameter were selected as mammospheres, manually counted and dissociated by incubation with 1:5 of 0.25% trypsin/EDTA. The mammosphere-forming efficiency (MFE) was calculated using the following equation: MFE = No. of spheres formed/No. of cells seeded × platting efficiency.

### 2.4. Colony Formation 

For the colony formation assay, MCF-7 cells (1000 cells/well) were grown on 6-well plates and maintained in a humidified chamber comprising 95% air and 5% CO_2_ at 37 °C for 14 days. Cells were then fixed with 3.7% paraformaldehyde at room temperature for 10 min and stained using crystal violet solution (0.2% crystal violet and 1X PBS) at room temperature for 30 min. Stained cells were washed with 1× PBS and air-dried at room temperature. The numbers of colonies were quantified using the Image J program (version- v1.53e). The survival fraction was calculated as the number of colonies counted/the number of cells inoculated × plating efficiency at 0 Gy. Colonies consisting of 50 or more cells were counted as clonogenic survivors. 

### 2.5. FACS Analysis for CD44/24 and ALDEFLUOR Assay

Cells were irradiated with the specific doses of radiation and then stained with anti-CD44-APC and anti-CD24-PE with their respective isotype controls, incubated for 40 min and analyzed on a *FACSCanto*™ flow cytometer (BD). To measure ALDH activity, cells were analyzed by an ALDEFLUOR assay kit (STEMCELL Technologies), following the manufacturer’s protocol. Flow cytometry was performed on a *FACSCanto*™ flow cytometer (BD), USA and analyzed by DIVA software (BD Biosciences, USA).

### 2.6. Immunoblotting

Protein extracts were prepared, and immunoblotting was performed, as described previously [15], using the following antibodies: Nrf2, Bach1, SNAIL and SLUG (Abcam, Burlingame, CA, USA); NQO1, HO1 (Santa Cruz, TX, USA), Keap1, SOX2 and KLF4, NANOG (Cell Signaling Technology, ((Danvers, MA, USA); BAX and BCL2 (Santa Cruz, Dallas, TX, USA) at 1:1000 and GAPDH (1:10,000, Sigma). Primary antibodies were detected using secondary antibodies (1:10,000, Bio-Rad), conjugated with HRP, and protein–antibody complexes were detected by the Substrate Detection Kit (Thermo Fisher, CA, USA). Densitometry was performed using the Image Lab and Image J software. GAPDH was used as loading control for whole-cell lysates. 

### 2.7. qRT-PCR 

Total RNA was extracted using TRIzol (Invitrogen). cDNA was synthesized using reverse transcription, followed by quantitative real-time PCR with SYBR Green Supermix (Life Technologies), using primers for Nrf2, Keap1, HO1, NQO1, SOX2, NANOG, KLF4 and GAPDH, which were used as the normalizing control. miR200a detection was carried out using the stem-loop method, as described previously [16]. The gene-specific primers used to perform real-time qRT-PCR analysis are listed in Table S1. 

### 2.8. Nrf2 Activity 

Nuclear protein fractions of irradiated MCF-7 cells were isolated using NE-PER Nuclear and Cytoplasmic Extraction Reagents (Thermo Fisher, MA, USA), according to the manufacturer’s instructions. Protein concentrations were assessed using the Bradford reagent (Bio-Rad). Nrf2 Transcription Factor Assay (Colorimetric) was performed using 20 mg of nuclear proteins (ab207223, Abcam) to detect nuclear Nrf2 and antioxidant responsive element (ARE) sequence binding at OD 450 nm, following the manufacturer’s instructions. 

### 2.9. Scratch Wound Assay 

MCF-7 cells were irradiated with specific doses of radiation and incubated for 24 h. The cells were scratched with a pipette tip to create wounds. Images were taken at different planes at 0 h and 24 h at 10× magnification. Percent cell migration was calculated as described in our previous paper [17].

### 2.10. ROS Detection

Detection of ROS was performed, as described previously [18]. Cells were treated with 1 μmol/L 2′,7′dichlorodihydrofluorescein diacetate (DCF-DA; Invitrogen, MA, USA) for 30 min, followed by a 1× PBS wash for 2 times. The reduced DCF-DA was oxidized by intracellular ROS and converted into fluorescent 2′, 7′-dichlorofluorescein (DCF). Fluorescent signals were detected by the *FACSCanto*™ flow cytometer (BD). A total of 10,000 cells were analyzed per sample.

### 2.11. Apoptosis and Cell Proliferation Assays

To perform Annexin V and propidium iodide (PI) staining, irradiated MCF-7 cells and mammospheres were trypsinized, washed with 1× PBS, centrifuged and stained with the Annexin V-FITC antibody (20 min, room temperature) and PI (0.02 mg/mL; Sigma, P4170). The percentage of apoptotic cells was evaluated using the *FACSCanto*™ flow cytometer (BD). A total of 1 × 10^4^ cells were recorded per condition in three independent experiments. In the cell proliferation assay, irradiated MCF-7 cells and mammospheres were trypsinized, washed with 1× PBS, centrifuged and stained with Ki67. The stained cells were analyzed using the *FACSCanto*™ flow cytometer (BD).

### 2.12. shRNA-Mediated Knockdown

To generate knockdown cell lines, MCF-7 cells were stably transduced in the presence of polybrene (5 μg/mL) with Nrf2 (sc-37030-V), shRNA lentiviruses and control scrambled shRNA particles-A (sc-108080) obtained from Santa Cruz Biotechnology, USA. Transduced cells were then selected with puromycin (2 μg/mL) for up to 4 weeks.

### 2.13. *In Vivo* Tumorigenicity Assay

Female SCID mice (6 to 8 weeks old; n = 5 per group) were maintained, according to the procedures and guidelines of the Institutional Animal Ethics Committee (NCCS). A total of 2 × 10^6^ MCF-7 cells (wild type/shNrf2) were injected subcutaneously into the mammary fat pads of female SCID mice along with a 1:2 ratio of growth-factor-reduced Matrigel (BD Biosciences). These mice were also injected with β-estradiol (Sigma-Aldrich, St. Louis, MO, USA) and observed for 2 months for the development of breast tumors. All the mice were euthanized, according to the institute’s ethical procedures, and tumors were collected for further analysis. The length and width of the tumors were measured using a vernier caliper and volumes were calculated using the following formula: Tumor volume = 1/2 (length × width^2^).

### 2.14. Bisulfite Sequencing and CpG Methylation Status

Genomic DNA was extracted using the DNeasy Tissue kit (QIAGEN, MD, USA). The EZ DNA Methylation Kit (Zymo Research, CA, USA) was used for sodium bisulfite conversion, according to the manufacturer’s protocol. Primers spanning two promoters of the Keap1 gene were designed using Methyl Primer Express (Thermo Scientific). Promoter 1 Forward: 5′- GAGTTTTGGYGGGGAATT-3′; Reverse: 5′-CCCTACCRCCTAAAACCAA-3′. Bisulfite-modified DNA (100 ng) was amplified in a PCR mix containing 0.4 µM of forward and reverse primer, HotStarTaq Master Mix Kit (Qiagen, Germany: 203445). Methylation status analysis was performed by Quantification Tool for Methylation Analysis (QUMA) software.

### 2.15. Statistical Analysis

One-way analysis of variance (ANOVA), followed by Tukey’s post hoc multiple comparisons tests by Prism software (GraphPad, San Diego, CA, USA), was used to analyze statistical significance. All the data values are presented as mean ± SE, reflecting the minimum of three independent determinations. Statistical significance was determined by comparing the treatments with untreated controls, and the significant differences are indicated as * *p* < 0.05, ** *p* < 0.01 and *** *p* < 0.001.

## 3. Results

### 3.1. Fractionated Doses of Radiation Selectively Increase E-BCSC Population While Decreasing M-BCSC Population

**Figure 1:**
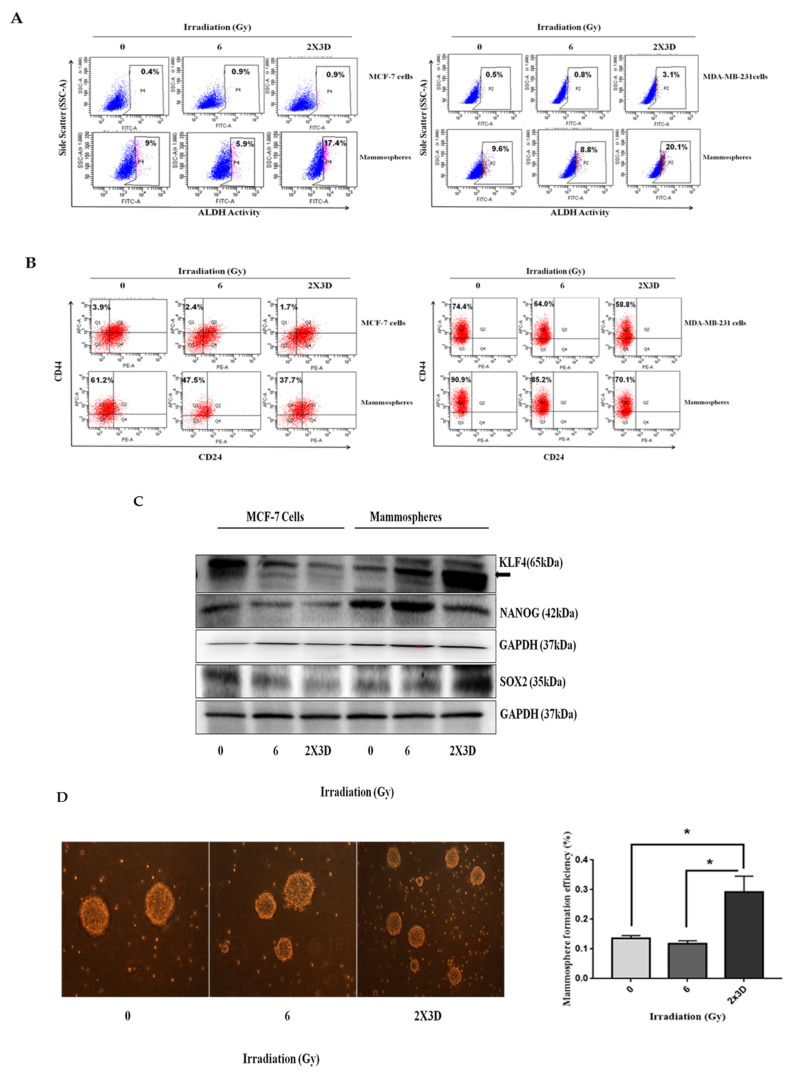
Effect of fractionated doses of radiation on breast cancer stem cell (BCSC) population induction and epithelial–mesenchymal transition (EMT). (**A**) BCSC population was identified in MCF-7 (left) and MDA-MB-231 cells (right) irradiated with a fractionated and acute dose of radiation by assessing ALDH activity and (**B**) CD44/CD24 markers using flow cytometry in MCF-7 (left) and MDA-MB-231 cells (right) after irradiation. (**C**) Expression of stem cell markers, i.e., NANOG and SOX2, was analyzed by Western blotting. (**D**) Phase-contrast images depict the effect of a fractionated and acute dose of radiation on sphere formation. All values are given as the mean ± SE, * *p* < 0.05, ** *p* < 0.01, *** *p* < 0.001 vs. control. All images are representative of three independent experiments.

Recent studies indicate that BCSCs exist in two phenotypes, i.e., epithelial (E-BCSC) and mesenchymal (M-BCSC), and BCSC plasticity plays a crucial role in future strategies for therapeutic resistance [19]. E-BCSCs characterized as ALDH^+^ population are proliferative, locate in the tumor’s hypoxic region and show the MET phenotype. On the other hand, M-BCSCs that express the CD44^+^/24^−^ phenotype are primarily quiescent, located on the invasive front and have the EMT phenotype. Previous studies have shown an increase in CD44^+^/24^−^ cells and high ALDH^+^ characteristics of tumor-initiating or cancer stem cells in breast tumors and established cell lines after irradiation [20,21,22,23]. In our study, fractionated irradiation with 2 Gy x 3 days of γ-rays increased the population of ALDH^+^ cells (Figure 1A) but decreased CD44^+^/24^−^ cells (Figure 1B) in MCF-7 and MDA-MB-231 cells and their corresponding mammospheres. Since mammospheres render an enriched BCSC population [24], we characterized these mammospheres by quantifying embryonic stem cell markers, SOX2 and NANOG. Compared to the MCF-7 cells, MCF-7-derived mammospheres express significantly high levels of SOX2 and NANOG, indicating the enriched BCSC population (Figure S1). Similar to ALDH activity, the expression of embryonic stem cell markers, i.e., SOX2 and NANOG in MCF-7 cells and mammospheres, and mammosphere formation efficiency (MFE) in MCF-7 cells was also increased upon exposure to fractionated doses of radiation (Figure 1C,D). Collectively, these results suggest that exposure of fractionated doses to radiation induces the E-BCSC phenotype in mammospheres, which may contribute to radioresistance and promote tumor recurrence.

### 3.2. Fractionated Doses of Radiation Induce Cellular Plasticity by Regulating EMT

**Figure 2:**
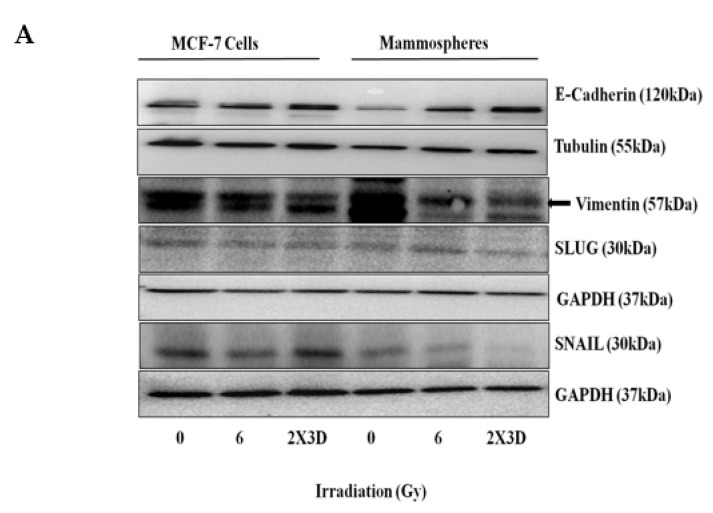

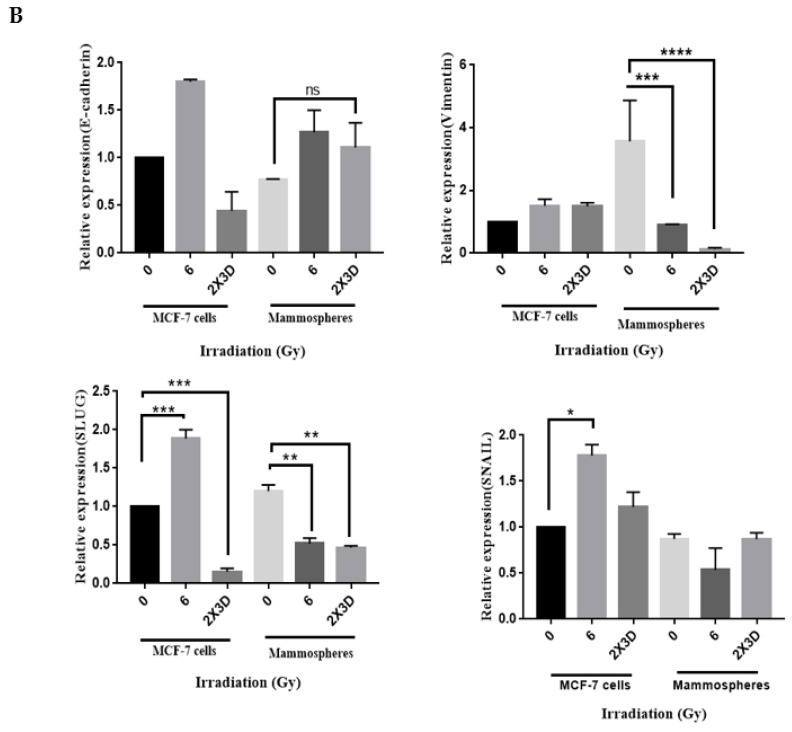
Effect of fractionated doses of radiation on EMT. (**A**) Expression of EMT markers, i.e., E-cadherin, Vimentin, SLUG and SNAIL, were analyzed by Western blotting and (**B**) qRT-PCR of E-cadherin, Vimentin, SLUG and SNAIL. GAPDH is used as loading control. All values are given as the mean ± SE, * *p* < 0.05, ** *p* < 0.01, *** *p* < 0.001, ****p < 0.0001 vs. control. All images are representative of three independent experiments.

An increase in E-BCSC signature in our study prompted us to further analyze the EMT markers. Fractionated irradiation caused the induction of MET, as levels of the epithelial marker E-cadherin were observed to be increased and the levels of mesenchymal markers Vimentin, SLUG and SNAIL were found to be decreased significantly only in BCSC-enriched mammospheres but not in MCF-7 cells (Figure 2A,B), thus inducing plasticity toward epithelial phenotype.

### 3.3. BCSCs with High ALDH^+^ Activity Display Radioresistance upon Exposure to Fractionated Irradiation

**Figure 3:**
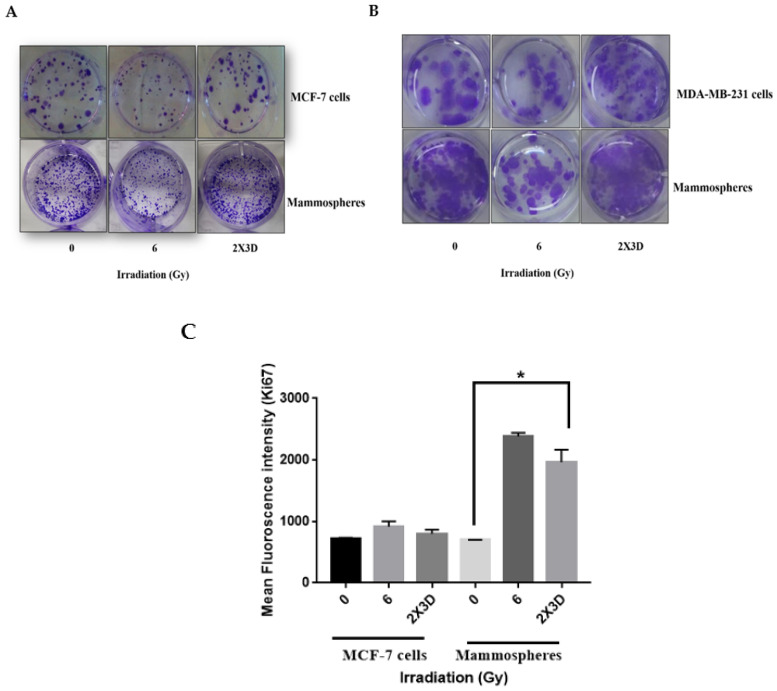

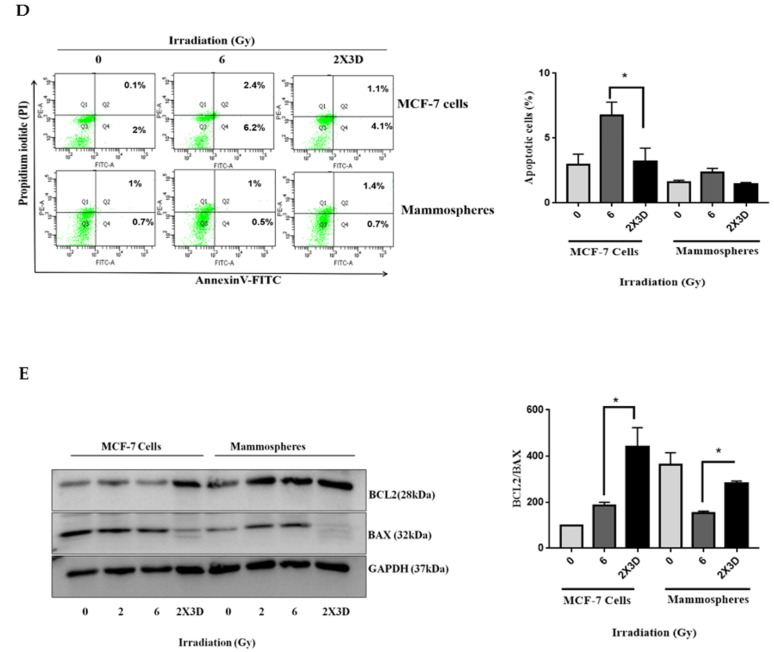
Fractionated doses of radiation enhance radiation resistance and reduce apoptosis in BCSCs. (**A**) Clonogenic assay was carried out for up to 14 days. The representative images show an increase in the colony formation of MCF-7 cells. (**B**) MDA-MB-231 and their corresponding mammospheres after irradiation with fractionated doses. (**C**) Cell proliferation was measured by analyzing the expression of Ki67 using flow cytometry. (**D**) The dot plots depict Annexin V-FITC and PI staining by flow cytometry. The horizontal (*x*) axis represents Annexin V-FITC and the vertical (*y*) axis represents PI staining. The bar graph represents the percentage of apoptotic cells as Annexin-V-FITC-positive cells (early apoptotic cells) and the percentage of Annexin-V-FITC- and PI-positive cells (late apoptotic cells). (**E**) BCL2 and BAX levels were analyzed by Western blotting. GAPDH was used as loading control. The representative bar graph shows the ratio of BCL2 and BAX. All values are given mean ± SE; * *p* < 0.05,; fractionated dose irradiation vs. acute irradiation.

Although controversial, previous findings suggest that BCSCs might be less sensitive to irradiation than cancer cells in in vitro assays [4,5]. We used a clonogenic cell survival assay to analyze the relative radioresistance of BCSCs. A single-cell suspension of MCF-7 cells was plated and irradiated with an acute dose (6 Gy) and fractionated doses (2 Gy × 3 days) of γ-rays. Our clonogenic survival assay demonstrated significantly higher radioresistance in MCF-7 and MDA-MB-231 cells and their corresponding mammospheres upon exposure to fractionated doses of radiation compared to controls (Figure 3A,B). Not only did the number of the colonies formed increase significantly after fractionated irradiation but also proliferative capacity, as indicated by Ki67 staining, was higher in these cells (Figure 3C). Ionizing radiation significantly increased the proportion of these CSCs and also showed enhanced proliferation shortly after treatment, further resulting in rapid tumor repopulation [25]. As there was an increase in the proliferation in cancer cells and mammospheres after fractionated irradiation, we further assessed apoptosis and the expression of anti- and proapoptotic genes, BCL2 and BAX. Although there was no significant change in the Annexin V^+^ apoptotic population in MCF-7 cells and mammospheres after fractionated irradiation compared to their respective controls (Figure 3D), a significant increase in the BCL2/BAX ratio was observed at the protein levels, further supporting radioresistance in these cells (Figure 3E).

### 3.4. The Emergence of Radioresistance Is Associated with High Migratory Potential and Tumorigenicity in Cancer Cells

**Figure 4:**
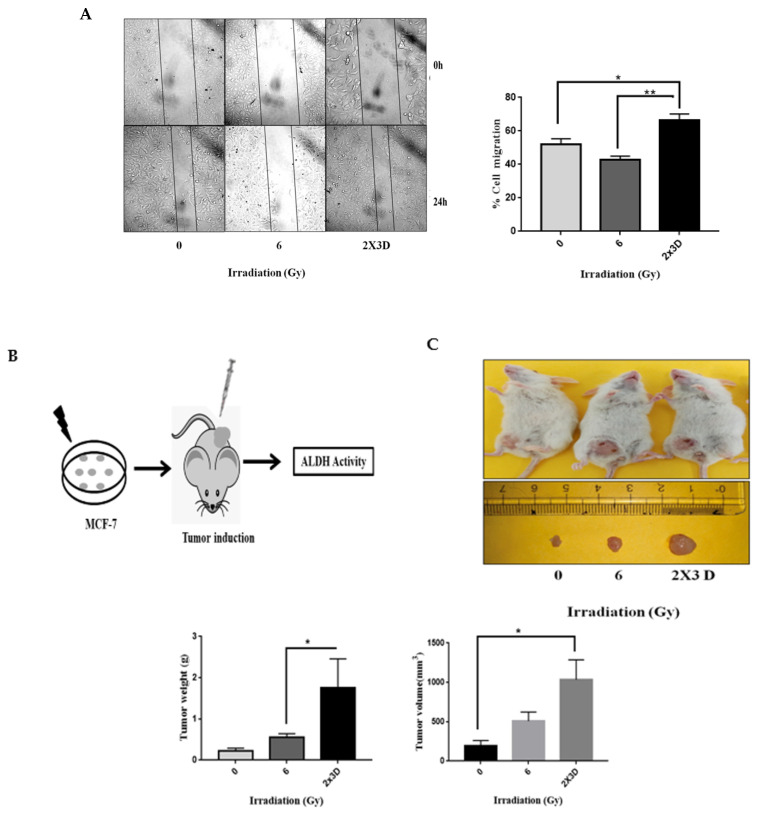

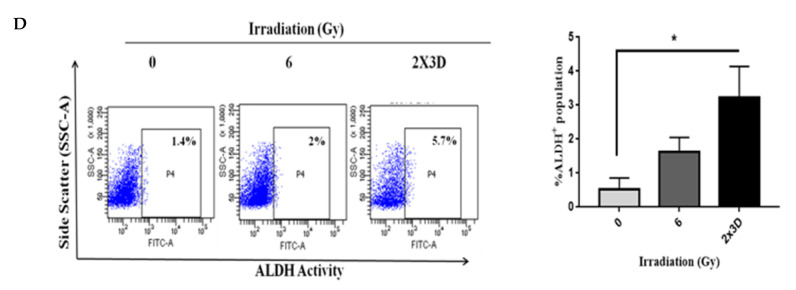
Fractionated doses of radiation enhance cell migration in vitro and tumor xenograft volume in vivo by increasing BCSC population. (**A**) Migration capacity was analyzed by scratch wound assay in confluent monolayers of irradiated MCF-7 cells and was expressed as % of gap closure of irradiated wells. (**B**) The flow diagram illustrates irradiated MCF-7 cells subcutaneously injected in SCID mice (n = 5). Tumors were dissected and dissociated in single cells, and ALDH activity was analyzed. (**C**) The image demonstrates isolated tumors. The bar graph represents tumor weight and volume of the xenograft tumors derived from MCF-7 control and irradiated cells. (**D**) ALDH activity was determined in isolated tumors using flow cytometry. All values are represented as mean ± SE. * *p* < 0.05; ** *p* < 0.01; vs. fractionated dose irradiation.

To analyze whether breast cancer cells irradiated with fractionated doses of radiation have functional characteristics of BCSCs, we examined their cell migration potential in vitro and tumorigenic properties in vivo. Compared to the controls and an acute dose, a significant increase in migration efficiency was observed in cells irradiated with the fractionated doses of radiation in the scratch wound assay (Figure 4A). Further, tumors in mice derived from MCF-7 cells irradiated with fractionated doses of radiation weighed significantly more than tumors derived from nonirradiated or acute-dose-irradiated MCF-7 cells (Figure 4B,C). Consistent with the in vitro results, analysis of the xenograft tumors derived from tumor cells irradiated with fractionated doses also showed enhanced ALDH activity (Figure 4D). Overall, these data demonstrate that fractionated dose exposure enhances migration potential in vitro and increases tumorigenicity by elevating the ALDH^+^ population in vivo.

### 3.5. Keap1-Nrf2 and not Bach1-Nrf2 Signaling Plays a Role in the Maintenance of Radioresistant ALDH^+^ BCSCs

**Figure 5:**
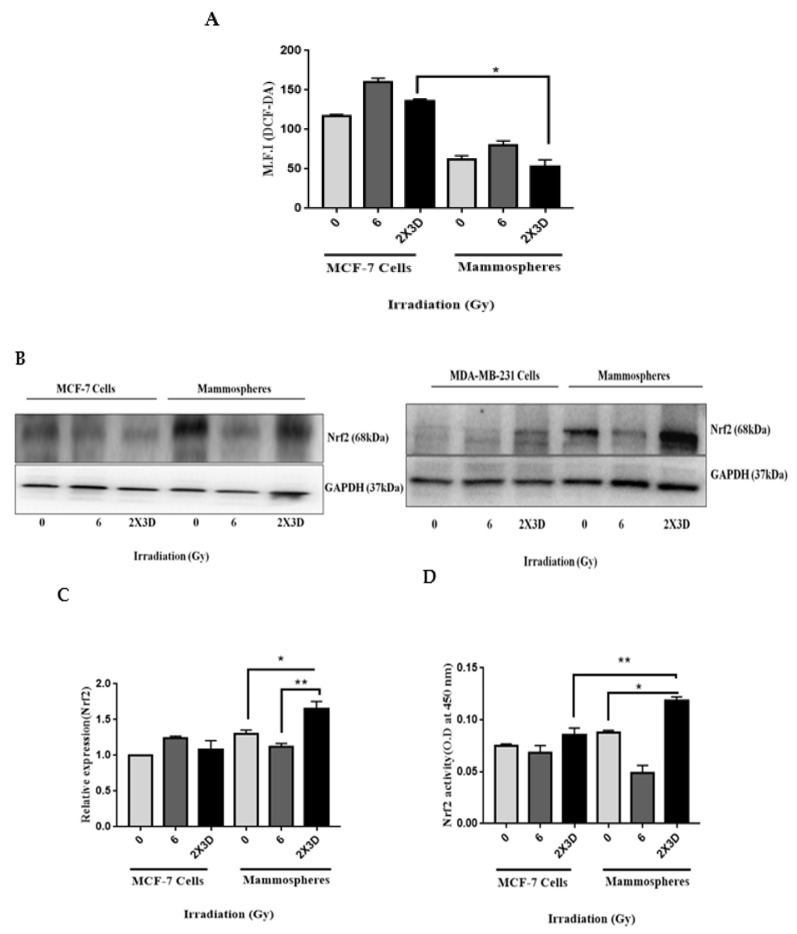

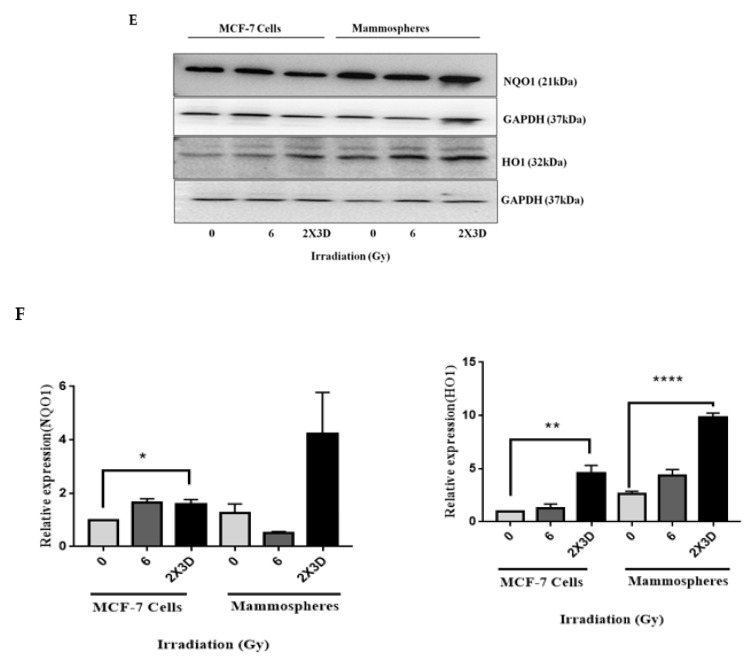

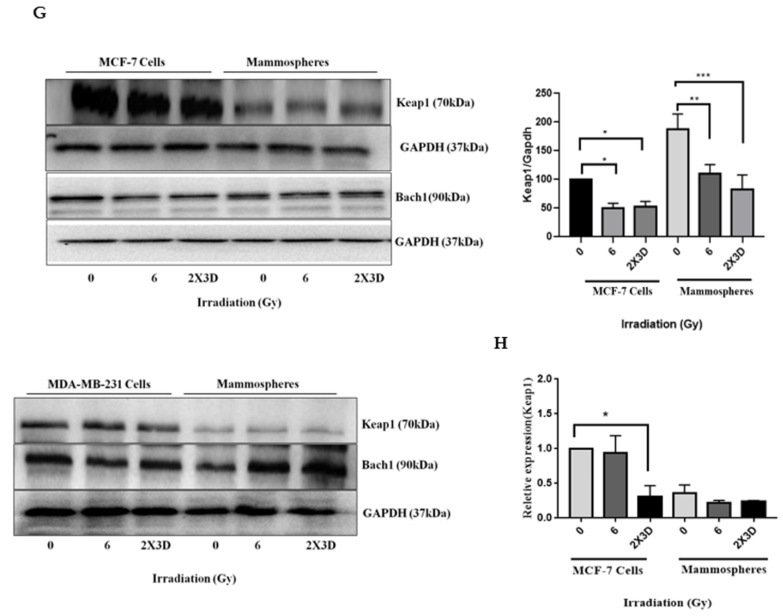
Fractionated doses of radiation generate low ROS and upregulate Nrf2 in BCSCs. (**A**) The bar graph represents ROS generation, assessed by DCF-DA staining using flow cytometry in MCF-7 cells and the corresponding CSC-enriched spheroids in control and irradiated cells (mean ± SE. * *p* < 0.05, fractionated-dose-irradiated MCF-7 cells vs. mammospheres). (**B**) Western blot analysis and (**C**) qRT-PCR illustrating the expression of Nrf2 in MCF-7 cell and MDA-MB-231 and their mammospheres. GAPDH served as loading control. (**D**) The bar graph represents the quantification of Nrf2 activity in irradiated MCF-7 cells and mammospheres. (**E**) The blots depict the Nrf2 targets HO1 and NQO1 by Western blotting. (**F**) The bar graph depicts the transcript levels of HO1 and NQO1 in irradiated MCF-7 cells and mammospheres by qRT-PCR. (**G**) Keap1 and Bach1 expression in irradiated MCF-7 cells (upper) and MDA-MB-231 (lower) and their mammospheres using Western blot analysis. (**H**) Transcript levels of Keap1 by qRT-PCR. GAPDH served as loading control. Mean from three independent experiments. All values are given mean ± SE. * *p* < 0.05, ** *p* < 0.01, *** *p* < 0.001; vs. fractionated dose irradiation.

Diehn et al. [5] showed that CSCs in breast tumors contain low ROS levels and enhanced ROS defenses compared to their nontumorigenic progeny, and these differences appear to be critical for maintaining stem cell function, which could contribute to tumor radioresistance. Previous studies have shown the involvement of Nrf2 in chemoresistance in BCSCs [12,13], hence we hypothesized that Nrf2 could also play a significant role in the radioresistance of BCSCs. We first determined the levels of ROS in MCF-7 cells and mammospheres irradiated with fractionated doses of radiation. We did not see any change in the ROS levels in these cells compared to their respective controls. However, an acute dose of radiation increased the levels of ROS in MCF-7 cells as well as in mammospheres (Figure 5A). Western blot and qRT-PCR analysis revealed that Nrf2 expression (Figure 5B,C), activity (Figure 5D), as well as its targets HO1 and NQO1 (Figure 5E,F), increased significantly when treated with fractionated doses of radiation. We observed a significant decrease in the expression of Keap1, and there was no change in the expression of Bach1, MCF-7 and MDA-MB-231 cells and their corresponding mammospheres irradiated with fractionated doses (Figure 5G,H), indicating that Keap1-mediated Nrf2 degradation is impaired, leading to the stabilization of Nrf2 and its nuclear accumulation [10,12]. The reduced level of ROS in our study could therefore be attributed to the activation of the antioxidant defense mechanism.

### 3.6. Inhibition of Nrf2 Concealed Radioresistance, Tumorigenesis and Induced Apoptosis via Reducing BCSC Population

**Figure 6:**
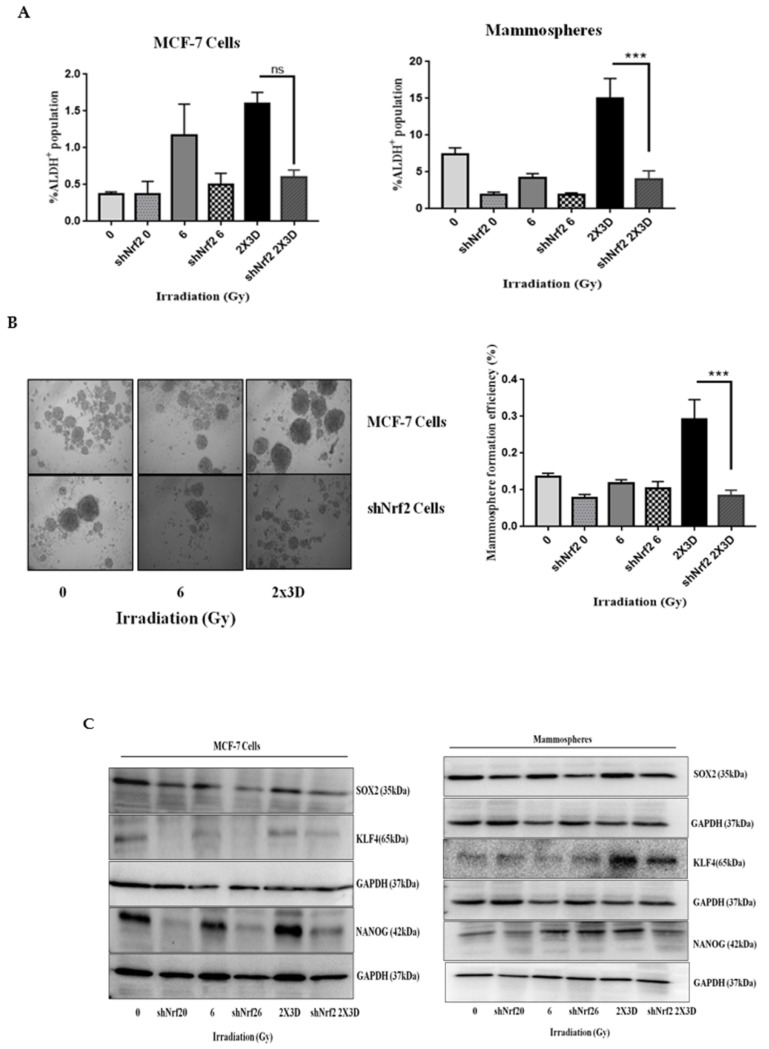

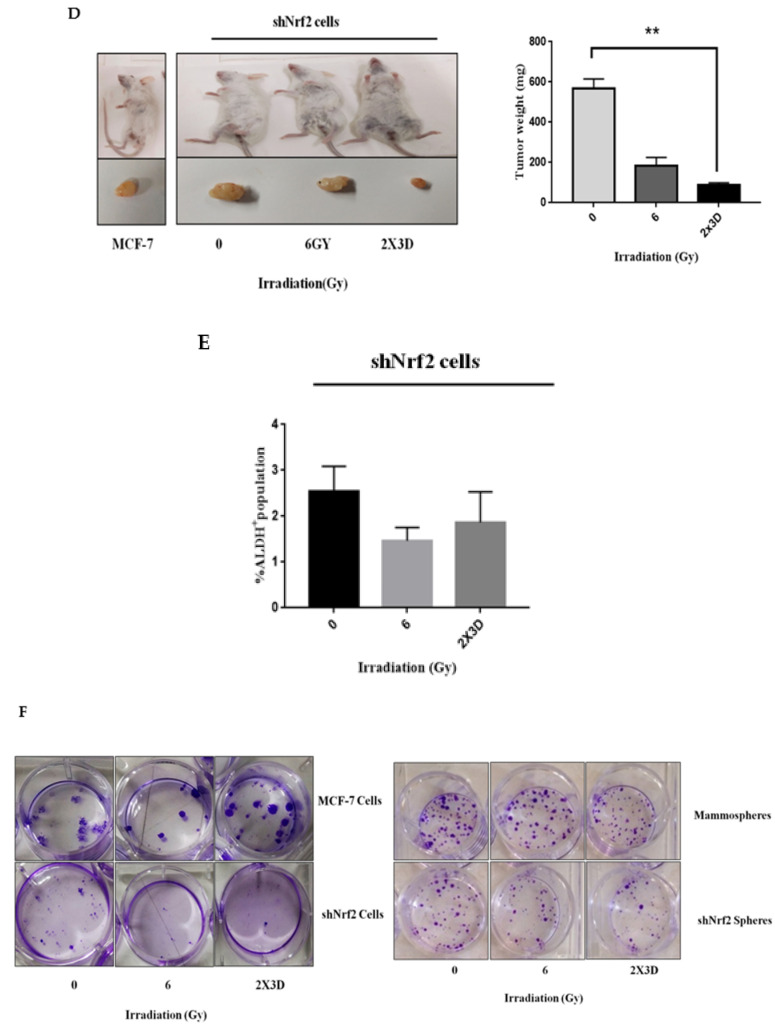

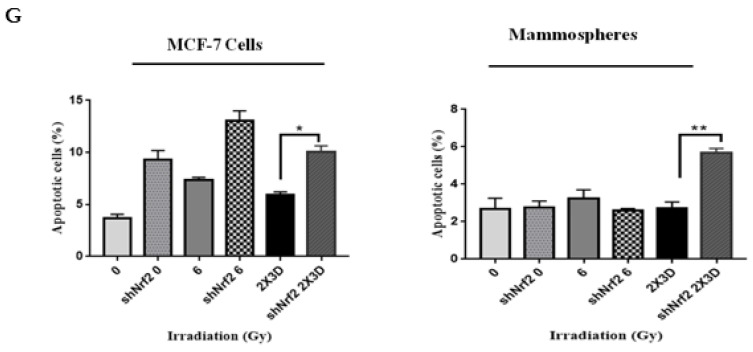
Inhibition of Nrf2 radiosensitizes breast cancer cells by inducing apoptosis and suppressing BCSC population after radiation treatment. (**A**) BCSC population measured by ALDH activity using flow cytometry in shNrf2 MCF-7 cells and mammospheres. (**B**) Phase-contrast images depict the effect of fractionated and acute doses of radiation on sphere formation in shNrf2 MCF-7 cells. The bar graph represents mammosphere formation efficiency for the same. (**C**) Expression of stem cell markers, i.e., SOX2, KLF4 and NANOG, was analyzed in shNrf2 MCF-7 cells and mammospheres by Western blotting, GAPDH is used as loading control. All values are given as the mean ± SE, *** *p* < 0.001 vs. fractionated-dose-irradiated shNrf2 cells. (**D**) The image demonstrates isolated tumors of the xenograft derived from shNrf2 MCF-7 control and irradiated cells. (**E**) ALDH activity was measured in shNrf2-derived tumors. (**F**) The representative images show a decrease in the colony formation of shNrf2 MCF-7 cells and mammospheres upon fractionated dose radiation treatment. (**G**) The bar graphs depict the percentage of apoptotic cells in shNrf2 MCF-7 cells and mammospheres. All values are given as the mean ± SE, * *p* < 0.05, ** *p* < 0.01; vs. fractionated dose irradiation shNrf2 cells. All images are representative of three independent experiments.

To further investigate the role of Nrf2 in radioresistance, Nrf2 was knocked down in MCF-7 cells (shNrf2). These cells showed a 55% reduction in Nrf2 transcripts levels (Figure S2). A 50% reduction in the population of ALDH^+^ cells was observed in Nrf2-knockdown mammospheres and MCF-7 cells after fractionated irradiation (Figure 6A). As a phenotypic effect, stable silencing of Nrf2 also resulted in the inhibition of mammosphere formation efficiency by two-fold in MCF-7 cells (Figure 6B). A reduction in the levels of SOX2, KLF4 and NANOG in these knockdown cells after irradiation indicated the role of Nrf2 in the suppression of BCSC population (Figure 6C). Tumorigenicity in SCID mice was decreased after injection of the irradiated Nrf2 knockdown cells. A significant decrease in tumor size (Figure 6D) as well as the percentage of ALDH^+^ population was observed in these tumors compared to the corresponding control (Figure 6E). Further, a reduction in clonogenicity (Figure 6F) and a significantly higher number of Annexin-V-/PI-positive cells were observed compared to their respective controls in shNrf2 mammospheres and MCF-7 cells irradiated with fractionated doses (Figure 6G). Thus, these results suggest that Nrf2 plays a crucial role in the acquisition of radiation resistance in BCSCs.

### 3.7. miR200a and not Promoter Methylation of Keap1 is Involved in Radioresistance of BCSC

**Figure 7:**
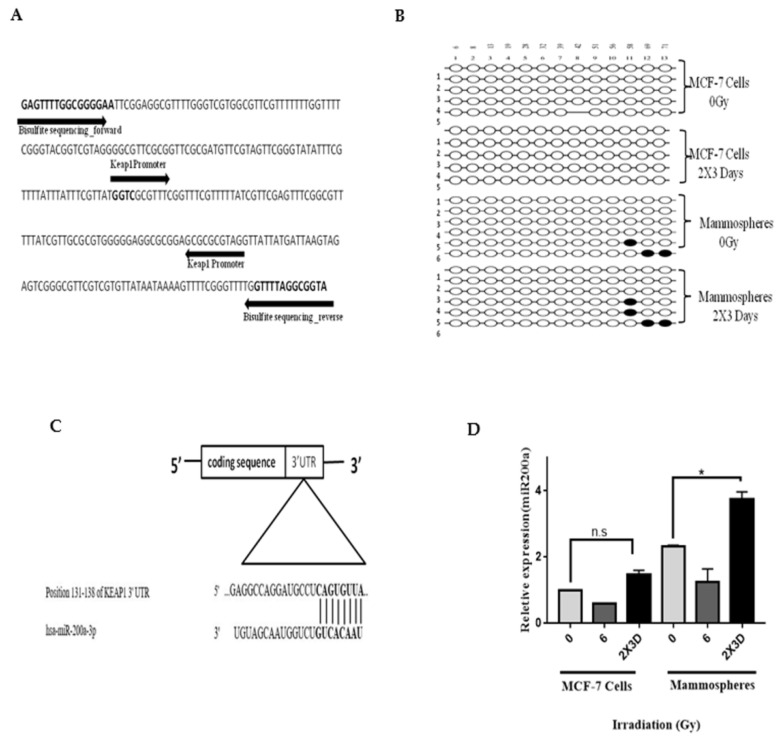
Promoter methylation and the role of miRNA200 in Keap1 regulation. (**A**) Primers’ design for bisulfite sequencing. The original genomic sequence of the Keap1 promoter region is shown. The Keap1 promoter contains 13 CpGs sites. (**B**) Keap1 promoter methylation by Quantification Tool for Methylation Analysis (QUMA) analysis: ○, unmethylated CpGs; ●, methylated CpGs. (**C**) Predicted binding sites between miR200a and Keap1 at 3′ UTR. (**D**) miR200a expression level in control and fractionated-dose-irradiated MCF-7 cells and mammospheres. All images are representative of three independent experiments. All values are given mean ± SE; * *p* < 0.05; vs. fractionated dose irradiation.

Since we observed a significant decrease in the expression of Keap1 at mRNA and protein levels, we further investigated its regulation at the epigenetic level, especially the methylation status of the Keap1 promoter by bisulfite sequencing [26]. We did not observe any change in the methylation status of the CpGs region in the Keap1 promoter, indicating that Keap1 promoter methylation may not be the key event in Nrf2 stabilization (Figure 7A,B). We next examined the role of the miR-200 family as it targets a conserved region in the Keap1 3′-UTR [27]. We observed no change in the expression of miR-141 but a significant increase in the expression of miR-200a, 1.4-fold in mammospheres and 1.85-fold in MCF-7 cells irradiated with the fractionated dose of radiation by RT-PCR (Figure 7C,D). Collectively, these results indicate that Keap1 downregulation could be due to increased miR200a; however, more studies are required to confirm the role of miR200a in this context.

## 4. Discussion

Radiation can induce cancer cell death by generating ROS and DNA damage; however, it is inefficient in targeting CSCs, which are largely responsible for therapy resistance, tumorigenesis and tumor recurrence [28,29,30]. Our study demonstrates that fractionated doses of radiation enhanced the E-BCSC marker ALDH^+^ and transcription factors of embryonic stem cells in BCSC-enriched mammospheres, indicating the E-BCSC phenotype, which is proliferative in nature. BCSC plasticity plays a crucial role in therapy resistance. BCSCs exhibit plasticity, which transitions between quiescent mesenchymal- (M-BCSCs) and proliferative epithelial-like (E-BCSCs) states [31]. An increase in E-BCSCs such as ALDH+ population and E-cadherin, indicative of MET, and a decrease in M-BCSCs such as CD44^+^/24^−^ population, the mesenchymal markers Vimentin, SNAIL and SLUG, demonstrated that fractionated doses of radiation increase the epithelial type of BCSCs [24,31,32]. Thus, these results support the notion that BCSC markers are not restricted to a particular population but change according to their plasticity based on the therapy. Hence, plasticity from M- BCSCs to E-BCSCs contributes to radioresistance. Since NANOG, SOX2 and KLF4 are essential for converting tumor cells into aggressive stem-like cells, an increase in the expression of these markers in our study after irradiation further supports the increased cancer stem cell population.

Emerging evidence indicates that Nrf2 plays a crucial role in CSC survival and resistance [33]. It is shown to be involved in chemotherapeutic drug resistance due to enhanced antioxidant capacity and detoxification of anticancer agents [14,34,35]. However, the involvement of the Nrf2-Keap1 axis in radioresistance of BCSCs is poorly understood. A strong association between low levels of ROS and enhanced antioxidant defense in BCSC radioresistance reported by Diehn et al. [5] prompted us to further investigate the role of Nrf2. Enhanced expression of Nrf2 and its downstream genes HO1 and NQO1 after irradiation in breast cancer cells and their corresponding mammospheres ascertains the involvement of Nrf2 in radioresistance. A recent report has shown that Nrf2 enhances ALDH^+^ E-BCSCs [24]. This supports our results, as we have observed a decrease in the ALDH^+^ E-BCSCs after Nrf2 inhibition. A decrease in embryonic stem cell markers, colony and sphere formation ability and reduced tumorigenicity after Nrf2 knockdown further indicate that Nrf2 is involved in the reprogramming process, and Nrf2 signaling is an important target for radiation resistance of BCSCs.

In the current study, Nrf2 appears to be regulated by Keap1 as we observed a decrease in the Keap1 levels with no change in the expression of either GSK-3β (Figure S3) [36] or Bach1. Additionally, as Bach1 binds to HO1 [10,11], an increase in the levels of HO1 in our study further confirms that Bach1 does not play a role in the regulation of Nrf2. Loss of Keap1 function is shown to mediate Nrf2 stabilization and is often associated with reduced drug sensitivity in several cancers [37,38,39]. A reduction in Keap1 expression with a concomitant increase in the expression of Nrf2 and its downstream targets HO1 and NQO1 clearly demonstrates the role played by Keap1 in Nrf2 regulation in the facilitation of acquired radioresistance. Hence, we tried to understand the mechanism of Keap1 regulation in this study.

Besides mutations through cysteine residues, epigenetic mechanisms, particularly the promoter hypermethylation [26], and miRNAs are the main regulators of Keap1. We did not see any change in the promoter methylation status of Keap1 after irradiation, which suggested that irradiation may regulate Keap1 post-transcriptionally rather than epigenetically. Hence, we further studied the role of the miR200 family as it is known to be involved in the regulation of Keap1. A significant increase in the transcript levels of miR200a indicates its role in the regulation of Keap1 in the radioresistance of BCSCs. Furthermore, reports from other studies have shown that miR200a suppresses the expression of transcriptional factors ZEB1/2 and inhibits the transition from the epithelial-to-mesenchymal phenotype [40]. This further strengthens and supports our studies where miR200a could be responsible for the inhibition of Keap1 as well as EMT in BCSC-enriched mammospheres.

In conclusion, the current study provides interesting insights into the mechanism by which fractionated doses of radiation increases radioresistance in the BCSC population. Our results indicate the enrichment of the E-BCSC phenotype. The regulation of Nrf2 in irradiated conditions occurs via the downregulation of Keap1 and not by GSK3β or Bach1. We provide mechanistic insight into the regulation of Keap1, possibly via post-transcriptional modification through miR200a and not via promoter methylation. Although the current study is limited to only the higher expression of miR200a, and given its potential for therapeutic purposes, additional mechanistic studies regarding its role in Keap1 inhibition and thus radioresistance is highly warranted. Nevertheless, alteration in the Nrf2-Keap1 pathway establishes relationships between radioresistance and BCSCs.

## Data Availability

The data presented in this study are available on request from the corresponding author.

## Supplementary Materials

The following are available online at https://www.mdpi.com/2073-4409/10/1/83/s1, Figure S1: Characterization of mammospheres. Figure S2: Nrf2 expression in Nrf2 knockdown cells. Figure S3: p-GSK3β levels in fractionated-dose-irradiated MCF-7 cells and mammospheres. Table S1: List of primers.
