# Supplementary material for: Keap1-Nrf2 Pathway Regulates ALDH and Contributes to Radioresistance in Breast Cancer Stem Cells
Source: Cells. 2021 Jan 6;10(1):83. doi: 10.3390/cells10010083 (PMC7825579; doi:10.3390/cells10010083)
Supplement: Supplementary file 1 [file cells-10-00083-s001.pdf]

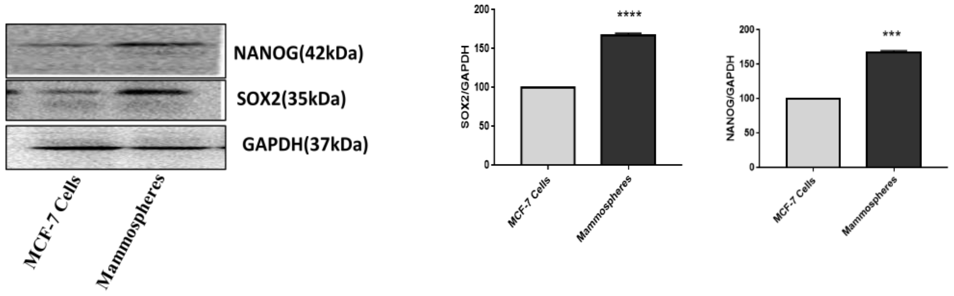

Figure S1. Characterisation of mammospheres. Expression of stem cell markers i.e. NANOG and SOX2 were analysed by western blotting GAPDH is used as loading control. All values are given as the mean ± SE, ***p<0.001 *vs* mammospheres. All images are representative of three independent experiments.

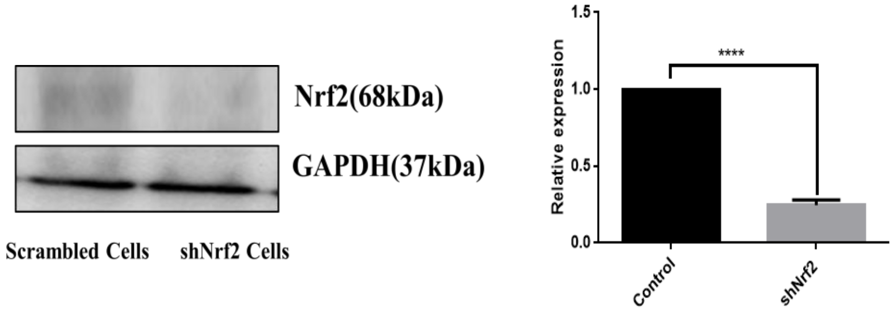

Figure S2. Nrf2 expression in Nrf2 knockdown cells. Expression of Nrf2 were analysed by western blotting and (B) RT-PCR. GAPDH is used as loading control. All values are given as the mean ± SE, ***p<0.001 *vs* shNrf2 cells. All images are representative of three independent experiments.

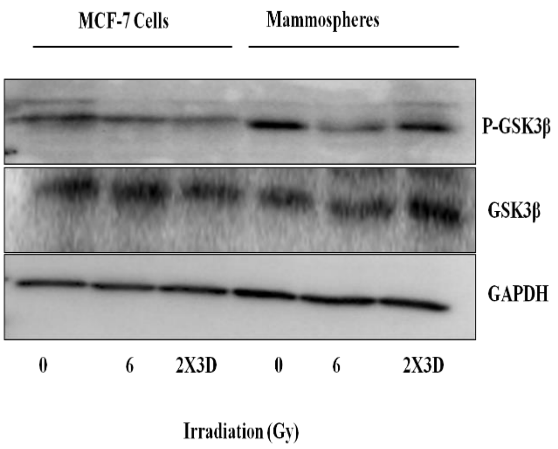

Figure S3. GSK3β levels in fractionated dose irradiated MCF-7 cells and mammospheres. Expression of p-GSK3β and GSK3β were analysed by western blotting. GAPDH is used as loading control.

Gene Name	Forward Primer	Reverse Primer
**Nrf2 (NEF2L2)**	5' -TCAGCGACGGAAAGAGTATGA- 3'	5'-CCACTGGTTTCTGACTGGATGT-3'
**Keap1**	5'-CTGGAGGATCATACCAAGCAGG-3'	5'-GGATACCCTCAATGGACACCAC-3'
**HO1**	5'-AAGACTGCGTTCCTGCTCAAC-3'	5'-AAAGCCCTACAGCAACTGTCG-3'
**NQO1**	5'-GAAGAGCACTGATCGTACTGGC-3'	5'-GGATACTGAAAGTTCGCAGGG-3'
**SOX2**	5'-GCCGAGTGGAAACTTTTGTCG-3'	5'-GGCAGCGTGTACTTATCCTTCT-3'
**NANOG**	5'-CCCCAGCCTTTACTCTTCCTA-3'	5'-CCAGGTTGAATTGTTCCAGGTC-3'
**KLF4**	5'-CCCACATGAAGCGACTTCCC-3'	5'-CAGGTCCAGGAGATCGTTGAA-3'
**CDH1(E-cadherin)**	5′-TGCCCAGAAAATGAAAAAGG-‘3	5′-GTGTATGTGGCAATGCGTTC-3′
**VIM (Vimentin)**	5′-CAGGAACAGCATGTCCAAATC-3′	5′-GGCAGCCACACTTTCATATTG-3′
**SNAI1(SNAIL)**	5′-AGCCTGGGTGCCCTCAAGATG-3′	5′-CTTGGTGCTTGTGGAGCAGGGAC-3′
**SNAI2 (SLUG)**	5′-GTCATACCACAACCAGAGATCC-3′	5′-GAGTATCCGGAAAGAGGAGAGA-3′

Table S1. List of primers
